# Olive (*Olea europaea*) Leaf Extract Induces Apoptosis and Monocyte/Macrophage Differentiation in Human Chronic Myelogenous Leukemia K562 Cells: Insight into the Underlying Mechanism

**DOI:** 10.1155/2014/927619

**Published:** 2014-04-06

**Authors:** Imen Samet, Junkyu Han, Lobna Jlaiel, Sami Sayadi, Hiroko Isoda

**Affiliations:** ^1^Graduate School of Life and Environmental Sciences, University of Tsukuba, Tennodai 1-1-1, Tsukuba, Ibaraki 305-8572, Japan; ^2^Faculty of Life and Environmental Sciences, University of Tsukuba, Tennodai 1-1-1, Tsukuba, Ibaraki 305-8572, Japan; ^3^Alliance of Research on North Africa, University of Tsukuba, Tennodai 1-1-1, Tsukuba, Ibaraki 305-8572, Japan; ^4^Laboratory of Environmental Bioprocesses, Biotechnology Center of Sfax, Sfax 3018, Tunisia

## Abstract

Differentiation therapy is an attractive approach aiming at reversing malignancy and reactivating endogenous differentiation programs in cancer cells. Olive leaf extract, known for its antioxidant activity, has been demonstrated to induce apoptosis in several cancer cells. However, its differentiation inducing properties and the mechanisms involved are still poorly understood. In this study, we investigated the effect of Chemlali Olive Leaf Extract (COLE) for its potential differentiation inducing effect on multipotent leukemia K562 cells. Results showed that COLE inhibits K562 cells proliferation and arrests the cell cycle at G0/G1, and then at G2/M phase over treatment time. Further analysis revealed that COLE induces apoptosis and differentiation of K562 cells toward the monocyte lineage. Microarray analysis was conducted to investigate the underlying mechanism of COLE differentiation inducing effect. The differentially expressed genes such as *IFI16*, *EGR1*, *NFYA*, *FOXP1*, *CXCL2*, *CXCL3*, and *CXCL8* confirmed the commitment of K562 cells to the monocyte/macrophage lineage. Thus our results provide evidence that, in addition to apoptosis, induction of differentiation is one of the possible therapeutic effects of olive leaf in cancer cells.

## 1. Introduction

Several advances against cancer have been recently achieved thanks to different therapeutic modalities, with radiation and chemotherapy being the most used so far. Although these therapies have been proven successful against some tumors, they are still highly toxic and nonspecific, since their primary mode of action is DNA damage, which results in severe adverse effects for normal cells [1]. Differentiation inducing therapy is therefore anticipated as a novel medical treatment that could reduce such adverse effects. This new concept which consists in forcing malignant cells to undergo terminal differentiation instead of killing them through cytotoxicity has so far gained a great interest especially for treating leukemia. Many compounds have been reported to induce differentiation of leukemia cells and some of them are already approved for clinical use [2]. Natural products have greatly contributed to cancer therapy and a rising interest is being attributed to the identification of new compounds from the plant resources with relevant effects against cancer development [3, 4]. Some of these compounds are now being used in clinical practice such as All-Trans Retinoic Acid. Recent basic research studies and observational epidemiologic studies strongly support that the disease-preventing effects of natural products are in part attributed to antioxidants, even though their efficiency in vivo needs more investigations [5].

Olive leaves contain many potentially bioactive compounds that may have antioxidant, antimicrobial, antihypertensive, antiviral, anti-inflammatory, hypoglycemic, neuroprotective, and anticancer properties [6–14]. Olive leaf has gained the rising interest of the scientific and industrial community due to its proved beneficial health properties and thus has emerged as commercially valuable nutraceuticals [15]. The primary constituents which are believed to contribute to the health benefits of olive leaves are Oleuropein, Hydroxytyrosol, as well as several other flavonoids, such as Verbascoside, Apigenin-7-glucoside, and Luteolin-7-glucoside [14, 16]. Oleuropein, the major constituent of olive leaves, has been shown to be a potent antioxidant. Its radical scavenging activity has been well documented [6, 17]. Oleuropein has been shown to inhibit the oxidation of low density lipoproteins in vitro and in vivo [18]. Jemai et al. have demonstrated that polyphenols recovered from olive leaf extracts, Oleuropein, Hydroxytyrosol, and Oleuropein aglycone, exhibited a pronounced hypolipidemic effect, reduced the lipid peroxidation process, and enhanced the antioxidant defense system in experimental atherogenic model [19]. Benavente-García et al., [17], studied the antioxidant activity of phenolic compounds from olive leaves and concluded that olive phenols may exhibit synergistic behavior in their radical scavenging capacity when mixed in the same proportions as occur in the olive leaf extract. Two recent studies have focused on the bioavailability of olive leaf phenolic compounds in human subjects and have come to the conclusion that Oleuropein is rapidly absorbed and metabolized to be mainly excreted as glucuronidated and sulfated Hydroxytyrosol, suggesting that olive leaf extract could exert benefits against oxidative stress-related processes in vivo [15, 20].

In the prior studies, olive leaf extract has been shown to exhibit an antitumor activity and to induce apoptosis pathways in cancer cells; little attention has been paid to its effect on the process of cancer cell differentiation. Particularly, olive leaf has been reported to exhibit an antileukemia effect by inducing apoptosis in the acute myeloid leukemia HL-60 cells [13, 21].

In this study, we investigate the effect of olive leaf of the most abundant Tunisian variety, Chemlali, on the human chronic myeloid leukemia K562 cells. Compared to the leukemia cell lines used in previous studies [13, 21], K562 cells can be regarded as stem-like cells thanks to their pluripotency [22] and are known for their strong resistance to chemical inducers [23].

We speculate that olive leaf extract may have anticarcinogenic property in K562 cells not only by inducing apoptosis but also by inducing the commitment of leukemia cells to the maturation process in order to progressively give an apparent normal cell life.

## 2. Materials and Methods

### 2.1. Preparation of Olive Leaf Extract

Collected olive leaves of Chemlali variety from the region of Sfax, Tunisia, were air-dried and ground with a mixer. Extraction was carried by ethanol 70% (1/10, w/v) in darkness for 2 weeks at room temperature. The mixture was then centrifuged and filtered using 0,45 *μ*m filter (Millipore, Japan) and stored at −80°C until it was used.

### 2.2. HPLC Analysis

Chromatographic analyses were achieved on an Agilent series 1260 HPLC-DAD instrument (Agilent, Waldbronn, Germany). The instrument includes a quaternary pump, an online degasser, an autosampler, and a thermostatically controlled column compartment. Chromatographic separation was carried out on a ZORBAX Eclipse XDB-C18 column serial number USNH027266 (4.6 mm I.D. × 250 mm × 3.5 *μ*m particle size). The elution conditions were as follows: mobile phase A (0.1% acetic acid in water) and mobile phase B (100% acetonitrile), flow rate of 0.5 mL/min, sample injection volume of 10 *μ*L, and operating temperature 40°C. The running gradient was as follows: 0–22 min, 10%–50% B; 22–32 min, 50%–100% B; 32–40 min, 100% B; 40–44 min, 100–10% B. Reequilibration duration lasted 6 min. The DAD detector scanned from 190 to 400 nm and the samples were detected at 254, 280, and 330 nm.

### 2.3. Cell Line and Culture Conditions

Human chronic leukemia cell line K562 was obtained from the Riken Cell Bank (Tsukuba, Ibaraki, Japan). Cells were cultured in RPMI 1640 medium (Gibco), supplemented with 10% heat-inactivated fetal bovine serum, and maintained at 37°C in a humidified incubator with 5% CO_2_. The cells were pass-cultured every 3 days and used for experiments after reaching the exponential growth phase.

### 2.4. Cell Proliferation Assay

Cell proliferation was investigated by MTT (3-(4,5-dimethylthiazol-2-yl)-2,5-diphenyltetrazolium bromide) assay. K562 cells were seeded in 96-well plates at 2.0 × 10^4^ cells/mL. After incubation for 24 h, olive leave extract diluted in medium was added at final concentrations of 50, 75, 100, 125, and 150 *μ*g/mL. Control cells were treated by ethanol at a final concentration of 0.3%. MTT was added after treatment for 24, 48, and 72 h and the resulting formazan was completely dissolved by 100 *μ*L of 10% sodium dodecyl sulfate (SDS) for 24 h. The absorbance was determined at 570 nm in a multidetection microplate reader (Powerscan HT, Dainippon Pharmaceutical, NJ, USA). Absorbance caused by the ability of the sample to reduce MTT or by its color was corrected using plates prepared in the same conditions in the absence of cells.

### 2.5. Cell Viability Assay and Cell Morphological Changes

The viability of COLE treated cells was measured using flow cytometry according to the manufacturer instructions. K562 cells were seeded in 6-well plates at 2.0 × 10^4^ cells/mL and treated the following day by 50, 100, and 150 *μ*g/mL of COLE diluted in medium and 0.3% ethanol in the case of the control. After incubation for the indicated time, treated cells were harvested, suspended in Guava ViaCount reagent, and allowed to be stained for at least 5 min in darkness. The cell number and viability were measured by Guava PCA flow cytometry (Guava Technologies, CA, USA). Morphological changes were detected by observation under a phase contrast microscope (Leica Microsystem).

### 2.6. Cell Cycle Analysis

The distribution of the cell cycle phases of treated and control cells was analysed by flow cytometry. Briefly, 2.0 × 10^4^ cells/mL of K562 cells were seeded in 6-well plates and treated by 100 and 150 *μ*g/mL of COLE diluted in medium. Control cells were treated with ethanol 0.3%. After the desired time of incubation, cells were harvested, washed twice with PBS, and fixed with 70% ethanol at 4°C for more than 12 h. The fixed cells were then centrifuged at 500 ×g for 5 min and washed with PBS twice. Cell cycle reagent (Guava Technologies) was added, and the cells were kept in darkness for 30 min at room temperature. The population of cells in each cell cycle phase was determined by a Guava PCA flow cytometry according to their DNA content.

### 2.7. Annexin V Assay

The induction of apoptosis in treated cells was determined by measuring the externalization of phosphatidylserine (PS) to the cell surface by flow cytometry. K562 cells were seeded in 6 well plates and treated with 50, 100 and 150 ug/mL of COLE diluted in medium. Control cells were treated with ethanol 0.3%. When reaching the desired treatment time, cells were harvested and stained with Guava Nexin Reagent. Then, cells were incubated for 20 min at room temperature in the dark and then acquired on the Guava PCA system.

### 2.8. Cell Differentiation Assay

Cell differentiation was assessed by flow cytometry by measuring the expression of CD11b and CD14 on the surface of K562 cells. Cells were seeded at 2.0 × 10^4^ cells/mL in 6-well plates and incubated for 24 h. The COLE diluted in medium was added at final concentrations of 50, 100, and 150 *μ*g/mL and ethanol at 0.3% in the case of control cells. After incubation, cells were harvested, washed twice with cold PBS, and adjusted to the same number 1.0 × 10^5^. The cells were then labeled with phycoerythrin conjugated anti-CD14, anti-CD11b, anti-CD41, and anti-glycophorin A for 30 min according to manufacturer's instructions. The stained cells were washed twice with cold PBS and resuspended in 500 *μ*L PBS for measurement.

### 2.9. Total RNA Isolation

K562 cells at a concentration of 2.0 × 10^4^ cells/mL were incubated for 24 h and then treated with 100 and 150 *μ*g/mL of COLE for 72 h. The control cells were treated with ethanol at final concentration of 0.3%. The cells were then collected and washed with PBS. DNA-free total RNA was isolated from the cells using Isogen reagent (Nippon Gene Co., Tokyo, Japan) following the manufacturer's instructions.

### 2.10. DNA Microarray Analysis

Microarray hybridization probes were generated from isolated RNA samples. Double-stranded cDNA was synthesized from 100 ng of total RNA using the GeneAtlas 3′ IVT Express Kit (Affymetrix, Inc.). Biotin-labeled aRNA was synthesized by in vitro transcription and purified. 10 *μ*g of purified aRNA was then fragmented using the GeneAtlas 3′ IVT Express Kit and was hybridized to the Affymetrix HG-U219 (Affymetrix) for 16 h at 45°C. The chips were washed and stained in the GeneAtlas Fluidics Station 400 (Affymetrix) and then imaged in the GeneAtlas Imaging Station (Affymetrix). The Partek Express software (Affymetrix) served for the data analysis by running comparisons of gene expression in treated and control cells based on mathematical algorithms. The generated data (significant fold change in gene expression) was then analyzed using the Pathway Studio Explore 1.1 software (Affymetrix).

### 2.11. Statistical Analysis

Data are presented as the mean ± SD of three independent experiments. Statistical analyses of changes, for each time and concentration point compared to the control, were performed using a paired two-tailed Student's *t*-test. A *P* value <0.05 was considered statistically significant.

## 3. Results

### 3.1. Composition of COLE

**Figure 1:**
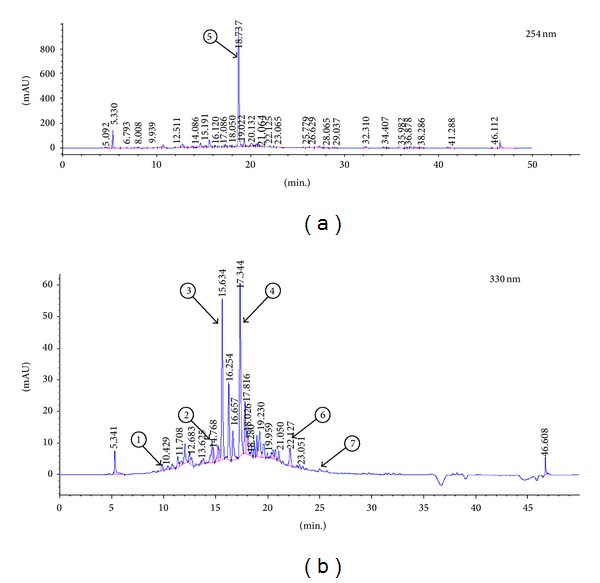
HPLC chromatogram of Chemlali Olive Leaf Extract (COLE) at (a) 254 nm and (b) 330 nm. Extraction was conducted with 70% ethanol. Peaks: 1, Hydroxytyrosol; 2, Verbascoside; 3, Luteolin-7-glucoside; 4, Apigenin-7-glucoside; 5, Oleuropein; 6, Luteolin; 7, Apigenin.

In order to identify and quantify the main compounds present in COLE, HPLC analysis was performed. Compounds were identified by comparing each peak's retention time with that of injected reference standards in the same chromatographic conditions. Only Oleuropein was detected at 254 nm while the two other phenylethanoids, Hydroxytyrosol and Verbascoside, as well as the flavonoids Apigenin and Luteolin and their glucoside forms were detected at 330 nm (Figure 1). The retention time (min) and the amount (mg/mL) of each detected compound are listed in Table 1. Oleuropein was the major compound in the extract, present at a concentration of 7.453 mg/mL. Luteolin-7-glucoside and Apigenin-7-glucoside were present at 0.536 and 0.529 mg/mL, respectively. The other detected compounds, Hydroxytyrosol, Verbascoside, Apigenin, and Luteolin, were present as traces.

The antioxidant activity of Chemlali Olive Leaf Extract was confirmed by the DPPH radical scavenging assay according to Enujiugha et al. [24]. Data showed that the radical scavenging activity of COLE increased in a dose dependent manner and that the extract concentration providing 50% inhibition of free radicals (IC50) was 0.6 mg/mL (data not shown).

### 3.2. COLE Inhibits the Proliferation of K562 Cells

**Figure 2:**
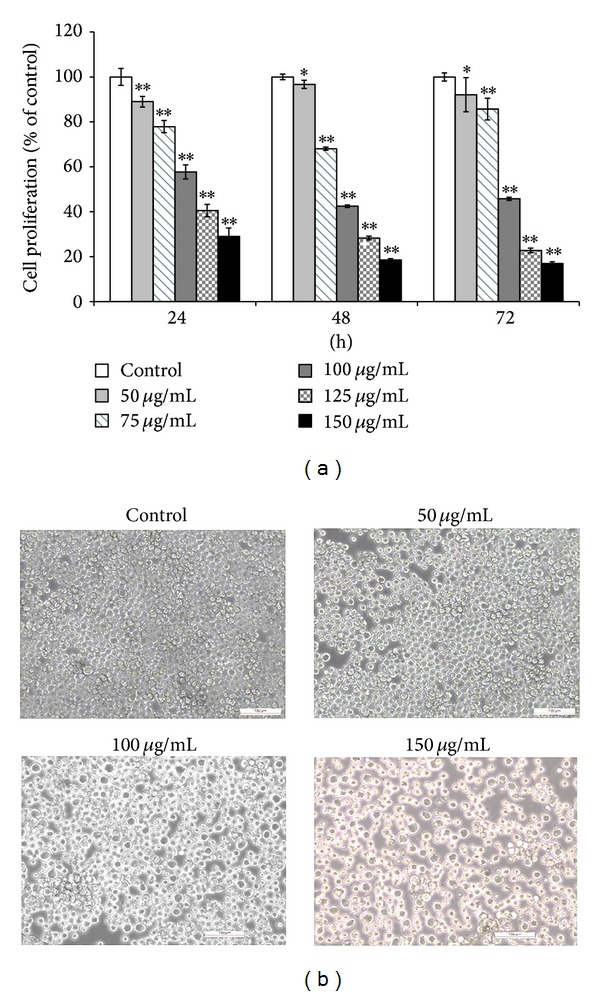
(a) Effect of Chemlali Olive Leaf Extract (COLE) on the proliferation of K562 cells. The cells were treated with various concentrations (50, 75, 100, 125, and 150 *μ*g/mL) of COLE for 24, 48, and 72 h. Cell proliferation was measured by MTT assay. Control represents cells treated with 0.3% ethanol in medium. Results are represented as the mean ± SD of three independent experiments. ∗, ∗∗: significantly different from the control (*P* < 0.05 and *P* < 0.01, resp.). (b) Morphological observations of K562 cells after treatment with various concentrations of Chemlali Olive Leaf Extract (COLE) for 72 h. Cells were observed under a phase contrast microscope at 100x magnification. Scale bars represent 100 *μ*m.

To evaluate the antileukemia effect of COLE on K562 cells, different concentrations from 50 to 150 *μ*g/mL were applied on K562 cells for the MTT assay. Treatment for 24, 48, and 72 h caused a significant decrease of the proliferation of K562 cells in a dose dependent manner. After 72 h of treatment with 150 *μ*g/mL of COLE, the cell proliferation was inhibited to 17% compared with the control cells (Figure 2(a)).

Observation of the morphological changes of K562 cells after treatment with the different concentrations revealed an increase in cell size especially in cells treated with 150 *μ*g/mL of COLE compared with control cells which kept the same morphology and cell size (Figure 2(b)).

### 3.3. COLE Inhibits the Growth Rate of K562 Cells

**Figure 3:**
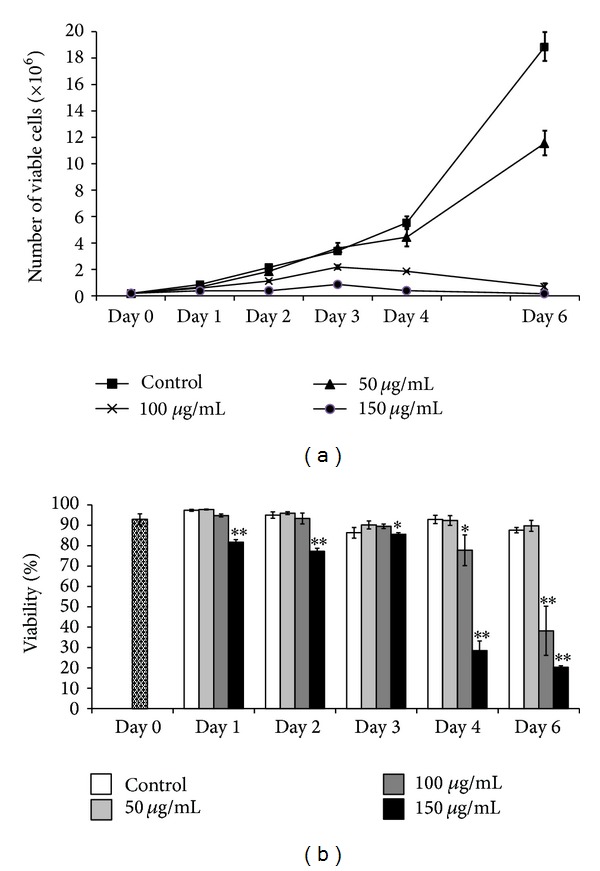
Effect of Chemlali Olive Leaf Extract (COLE) on the cell number and viability of K562 cells. (a) Number of viable cells after treatment with COLE after incubation up to 6 days. (b) Percentage of viability in K562 cells treated with COLE. K562 cells were treated at a final concentration of 50, 100, and 150 *μ*g/mL of COLE and incubated for different periods. Cell number and cell viability were measured by flow cytometry. Control represents cells treated with 0.3% ethanol in medium. Results are represented as the mean ± SD of three independent experiments. ∗, ∗∗: significantly different from the control (*P* < 0.05 and *P* < 0.01, resp.).

To understand if the antiproliferative effect was led by cell death or growth inhibition, we determined the cell number and cell viability after treatment with COLE by flow cytometry. The numbers of K562 cells treated with 100 *μ*g/mL and 150 *μ*g/mL were significantly less than the number of control cells. K562 cells treated with COLE exhibited a slow cell growth compared to the control (Figure 3(a)).

Results (Figure 3(b)) showed that treatment with 150 *μ*g/mL of COLE caused a slight decrease of the viability of K562 cells during the first 3 days with keeping more than 80% of viable cells. Then the decrease became drastic and only 20% of live cells remain at the 6th day of treatment by the same concentration. The viability of cells treated with 100 *μ*g/mL of COLE started to decrease significantly from the 4th day, while treatment with 50 *μ*g/mL did not affect the cell viability during the whole period. Taken together, these results suggested that the antiproliferative effect of COLE at the concentrations of 100 *μ*g/mL and 150 *μ*g/mL might be conducted in part by inhibiting the growth rate and to a lesser extent by causing cell death.

### 3.4. COLE Modulates Cell Cycle Progression in K562 Cells

Since COLE reduced the growth of K562 cells, we investigated its effect on the cell cycle progression. During the 1st and 2nd day, K562 cells treated with 100 and 150 *μ*g/mL were arrested at G0/G1. During the following days, results showed a significant increase of G2/M population in cell treated with 100 and 150 *μ*g/mL of COLE. In the case of cells treated with 150 *μ*g/mL of COLE, this increase was associated with a significant decrease of G0/G1 cell population throughout the 3rd and 4th days of treatment (Table 2).

### 3.5. COLE Induces Apoptosis in K562 Cells

**Figure 4:**
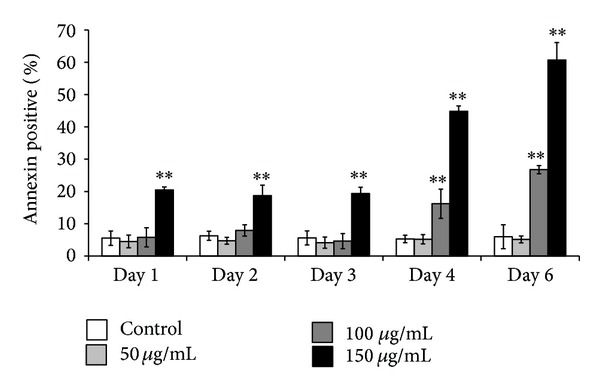
Induction of apoptosis in K562 cells treated with Chemlali Olive Leaf Extract (COLE). Cells were treated with 50, 100, and 150 *μ*g/mL of COLE and incubated for different time. At the indicated time, K562 cells were stained with Annexin V and analyzed by flow cytometry. Control represents cells treated with 0.3% ethanol in medium. Results are represented as the mean ± SD of three independent experiments. ∗, ∗∗: significantly different from the control (*P* < 0.05 and *P* < 0.01, resp.).

The decrease in cell viability (Figure 3(b)) caused by COLE treatment prompted us to investigate if the extract induces apoptosis in K562 cells. Apoptosis is an important and active regulatory pathway of cell growth and proliferation resulting in some characteristic physiological changes. Among these, externalization of phosphatidylserine (PS) is easily detected by flow cytometry after binding to the labeled Annexin V. The results indicated an increase in Annexin V positive cells from the 1st day of incubation with 150 *μ*g/mL of COLE in comparison with control cells (Figure 4). The percentage of Annexin V cells remained stable during the first 3 days of treatment with 20% of total treated cells and interestingly increased in the 4th and 6th days with 45% and 60%, respectively. Treatment with 100 *μ*g/mL caused the apparition of apoptotic cells from the 4th day of incubation with a proportion of 16.2%. This population increased in the 6th day up to 26.7%. No significant difference in apoptotic cells amount was detected when cells were treated with 50 *μ*g/mL until 6 days of incubation.

### 3.6. Impact of COLE on the Differentiation Capacity of K562 Cells

**Figure 5:**
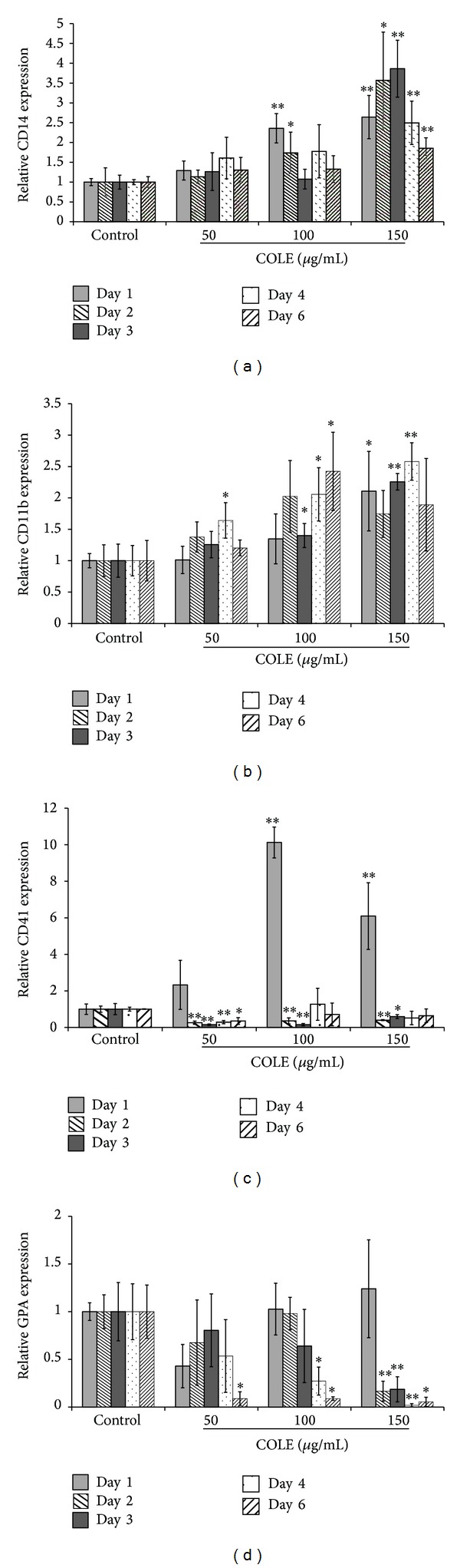
Expression of differentiation markers on K562 cells treated with Chemlali Olive Leaf Extract (COLE) up to 6 days. Cells were treated with 50, 100, and 150 *μ*g/mL of COLE and incubated for different periods. Control cells were treated with ethanol at a final concentration of 0.3%. Expression of cell surface markers was detected by flow cytometry. (a) Expression of CD14 (monocyte/macrophage specific marker). (b) Expression of CD11b (granulocyte/monocyte specific marker). (c) Expression of CD41 (megakaryocyte specific marker). (d) Expression of glycophorin A (GPA) (erythrocyte specific marker). Results are represented as the mean ± SD of three independent experiments. ∗, ∗∗: significantly different from the control at *P* < 0.05 and *P* < 0.01, respectively.

The reduction in cell growth, as well as the morphological changes observed in COLE treated cells, led us to think about the differentiation assessment. K562 cells are pluripotent malignant cells that spontaneously differentiate along erythroid, macrophage, and megakaryocyte lineages [22]. Treated cells were harvested and analyzed for the expression of lineage differentiation markers. The analysis was performed by flow cytometry at different days on the monocyte/macrophage marker CD14, on the granulocyte marker CD11b, on the erythrocyte marker GPA and on the megakaryocyte marker CD41. The results showed a significant increase in the expression of CD14 marker from day 1 until day 6 in the cells treated with 150 *μ*g/mL, suggesting the commitment of K562 cells to the monocyte/macrophage lineage (Figure 5(a)). A slight increase of the expression of CD11b was also detected at this concentration (Figure 5(b)). Treatment with 100 *μ*g/mL showed a gain in the expression of CD14 on the 1st and 2nd day and an augmentation of CD11b expression from the 3rd day of treatment. Treatment with 50 *μ*g/mL did not show any significant effect on the differentiation markers CD14 and CD11b. Even after 6 days of treatment with 150 *μ*g/mL of COLE, we could not detect the erythroid marker at all the tested concentrations (Figure 5(d)). However, an instant increase in the megakaryocytic marker was detected on the 1st day of treatment, followed by a drastic decrease, lower than the control, during the following days (Figure 5(c)).

### 3.7. Gene Expression Profile of COLE Treated K562 Cells

To further elucidate the mechanism by which COLE induces apoptosis and differentiation of K562 cells, we investigated the changes in gene expression profiles in treated cells using HG219 GeneChip array. Microarray analysis was performed on K562 cells at the 3rd day of treatment based on the observation that such timing generated a significant expression of the differentiation markers. Genes with more than 1.5-fold change in expression levels between control and 150 *μ*g/mL of COLE treated cells were classified into categories according to the cell biological processes. According to the GO analysis results (Tables S1 and S2 in Supplementary Material available online at http://dx.doi.org/10.1155/2014/927619/), COLE treatment induced the upregulation of genes involved in hematopoiesis such as* CTNNB1*,* SH2B3*,* CIAPIN1*,* RPA1*, and genes implicated in cell differentiation and its regulation such as* MCL1*,* CTNNB1*, and* CCNE1*. Interestingly, genes related to myeloid cell differentiation, such as* IFI16*,* ACIN1*, and* CASP8*, and particularly genes engaged in monocyte differentiation were represented in the upregulated categories. Moreover, an increased expression of genes involved in chemotaxis such as* NUP85*,* HRAS*,* IL8*,* CXCL2*, and* CXCL3* and genes related to cytokine production was also detected. Among upregulated genes* AP1G1*,* RABEP1*,* RAB5C*,* RAB21*,* EPS15L1*, and* CORO1C* were also found involved in the process of endocytosis and phagocytosis as well as genes related to protein transport and genes implicated in cell adhesion such as* ICAM3*,* HSPB11*, and* PNN*.

Conversely, the erythrocyte differentiation categories were represented in the list of the decreased genes (Table S2) including* GYPA*,* HBE1*,* FECH*, and* ALAS2*, which is consistent with the decreased expression of erythrocyte differentiation marker in the flow cytometry results (Figure 5(d)).

Analysis of the microarray data showed the upregulation of several proapoptotic genes and genes involved in the regulation of mitochondrial membrane permeability such as* CASP6*,* CASP8*,* DFFA*, and* BID*. On the other hand, expression of genes negatively regulating the apoptosis and those inhibiting the caspase activity was significantly decreased such as* IGF1R*,* HSPA5*, and* BCL2*.

Among the upregulated genes, we found those related to the NF-kappaB cascade as well as to the MAPKKK cascade and the Wnt receptor signaling pathway.

Categories related to cell cycle regulation and cell proliferation were represented by both upregulated and downregulated genes. Finally, some transcription factors were also downregulated, such as* FOXP1*, whereas others were upregulated like* EGR1* and* NFYA*.

The list of selected genes is presented in Table 3. From this list, it is clear that the differentially expressed genes after treatment with 100 and 150 *μ*g/mL have mostly the same tendency, which could validate the obtained results.

## 4. Discussion

Previous reports had indicated that olive leaf extract induces apoptosis in the human leukemic cell lines HL-60 and Jurkat cells [13, 21]. In this study we give evidence that COLE exhibits its antileukemia effect by both inducing apoptosis and promoting differentiation of the multipotent human leukemia K562 cells.

Induction of differentiation, as well as apoptosis, has been frequently reported to be associated with a loss of proliferative capacity of the cells. It has been reported that olive leaf extract inhibits the proliferation of some cancer cell lines such as B16 melanoma cells, HL-60 leukemia cells, the breast cancer MCF-7 cells, and the glioblastoma cells [12, 25–27]. Our results indicate that COLE inhibits the proliferation of K562 cells in a dose dependent manner (Figure 2(a)). However, despite the drastic decrease in cell proliferation, the viability of cells treated with the highest concentration of COLE (150 *μ*g/mL) remains relatively high (80%) for up to 3 days of treatment (Figure 3(b)).

Since it is well established that the arrest of the cell cycle progression allows the cells to follow other processes such as apoptosis and differentiation, we then explored the effect of COLE on the cell cycle distribution (Table 2). Olive leaf extract has been shown to cause cell cycle arrest at G0/G1 phase in both the breast cancer MCF-7 cells and the melanoma B16 cells [12, 25]. However, the treatment times applied in both of these studies were limited to 48 h and 18 h, respectively. In this study, COLE treated cells were arrested at G0/G1 on the 1st and 2nd day of treatment, which is consistent with the reported studies. Prolonged incubation with COLE showed an arrest of cell cycle at G2/M phase (3rd and 4th day of incubation). The microarray analysis, performed for the 3rd day of treatment, revealed a significant increase of* CHEK2* gene expression in COLE treated cells. The encoded protein regulates the cell cycle checkpoint arrest through the inhibition of the activity of CDC25A, CDC25B, and CDC25C [28]. CDC25 proteins trigger the entry into mitosis at different points of the cell cycle by activating the Cdk-cyclin complexes. CDC25A acts early in the cell cycle, regulating the G1/S transition, whereas CDC25B and CDC25C act at G2/M [29]. Interestingly, our results showed a decrease in the expression of* CDC25C* gene in COLE treated cells associated with an increase in the expression of* CDC25A*, which may explain the cell cycle arrest at G2/M phase observed at the 3rd day of treatment with COLE.

Treatment with COLE increased the expression of CD14 on the cell surface of treated cells indicating the differentiation of K562 cells toward the mono-/macrophage lineage (Figure 5(a)). The increase in the percentage of cells positive for CD11b (Figure 5(b)) confirms this hypothesis. In fact, CD11b is expressed on both monocytes and granulocytes. Thus, together with the increase of CD14 expression, the expression of CD11b suggests that treatment with COLE promotes the commitment of K562 cells into the monocyte/macrophage lineage at the expense of the granulocytic differentiation. The instant increase of the expression of CD41 on the 1st day of treatment and its absence during the following days (Figure 5(c)) could be consistent with monocyte differentiation instead of megakaryocyte differentiation since it has been reported that *α*IIb promoter, which initiates the transcription of CD41, is transcriptionally active in pluripotent myeloid progenitors, in early stages of erythropoiesis, and all along the megakaryocytic differentiation as well as, to a lesser extent, in the early stages of myelomonocytic differentiation and the late stages of erythropoiesis [30]. This instant increase could be also explained by the phenomenon of lineage conversion, where some hematopoietic progenitors can be converted into lineages other than their own by the ectopic expression of some transcription factors [31]. In this regard, it was previously reported that common lymphoid progenitors, megakaryocyte erythroid progenitors, early B cells, and early T cells could be converted to the granulocyte/monocyte lineage by the induction of C/EBP*α* activity [32]. Microarray results support the absence of megakaryocyte differentiation in the 3rd day of treatment. In fact, data showed a decrease in the expression of genes related to megakaryopoiesis such as the essential regulator of platelet release* NFE2* and its main candidate target gene* TUBB1* (encoding the megakaryocyte and platelet specific isoform of *β*-tubulin (*β*1)) [33] as well as the* BACH2* transcription factor reported to be induced during megakaryocyte differentiation [34].

Interestingly, results of microarray analysis (Table 3) showed the upregulation of several genes already reported as key players in monocyte/macrophage differentiation in the COLE treated cells. Among these genes, the expression of* IFI16* was markedly increased. Constitutively expressed in lymphoid cells,* IFI16* was shown to be associated with the differentiation of human myeloid cells in response to interferon gamma [35]. Moreover,* IFI16* has been selectively expressed during the differentiation of myeloid progenitor cells along monocytic lineage, while it is downregulated during the commitment toward the granulocytic or erythroid lineage, suggesting its potential involvement in the differentiation and maturation of the monocytic lineage [36, 37]. Gene encoding for* EGR-1*, a positive modulator of macrophage differentiation, was also significantly upregulated.* EGR-1* is known to dictate development of myeloid progenitors along the macrophage lineage at the expense of development along other lineages [38, 39]. Dauffy et al., [40], reported that* IFI16*-enforced expression in myeloid progenitors induced the expression of* EGR-1* and conducted the macrophage differentiation in the absence of the macrophage colony stimulating factor (M-CSF). This suggests that* EGR-1* could be regulated by the expression of* IFI16*. Treatment with COLE increased as well the expression of* NFYA* encoding the A subunit of the nuclear transcription factor Y whose synthesis was previously reported during the terminal differentiation of monocytes to macrophages [38, 41]. The downregulation of* FOXP1* in COLE treated K562 cells is consistent with monocyte/macrophage differentiation since it has been demonstrated that the expression of* FOXP1* was markedly decreased in monocyte-induced differentiation of HL-60 cells as well as in human peripheral blood monocytes and that the overexpression of* FOXP1* prevented the morphologic macrophage-like differentiation [42, 43].

Monocytes and macrophages have several functional characteristics including cell adhesion, migration, chemotaxis, and phagocytic activity. Interestingly, an enhanced expression of the chemokines genes* CXCL8* (*IL8*),* CXCL2*, and* CXCL3* was found in COLE treated cells. IL-8 production has been observed in vitro in a wide variety of cells including monocytes, T lymphocytes, neutrophils, vascular endothelial cells, dermal fibroblasts, keratinocytes, hepatocytes, and human gastric cancer cells, while the production of CXCL2 and CXCL3 was only described in monocytes, fibroblasts, and endothelial cells [44, 45]. Another gene related to chemotaxis was upregulated: NUP85 which is an essential component of the nuclear pore complex and was reported to be involved in CCR2-mediated chemotaxis of monocytes [46].

COLE treatment also enhanced the expression of some genes related to phagocytosis: AP1G1 and Rab proteins related genes. AP1G1 is a subunit of clathrin-associated adaptor protein complex 1 whose role has been recently highlighted for efficient phagocytosis at an early stage of phagosome formation since it participates in the extension of the phagocytic cup [47, 48]. The small GTPases Rab are key regulators of intracellular membrane trafficking. Recent studies have demonstrated that several RAB proteins play an important role in phagocytosis. RAB proteins such as RAB11, Rab5, and Rab 21 participate in the phagosome formation and maturation and are necessary for the phagocytic activity [48–51].

Consistently with the increase in Annexin V positive cells (Figure 4), treatment with COLE upregulated some proapoptotic genes such as* CASP6*,* CASP8*,* DFFA*, and* BID* and downregulated the apoptosis suppressor* BCL2* as well as the caspase inhibitors genes (Table 3). Olive leaf was previously shown to induce morphological changes that are characteristic of apoptosis in leukemia cells, but the mechanisms underlying this effect were not well investigated [13, 21]. The increase in the proportion of apoptotic cells observed from the 4th day of treatment with COLE could be explained by the programmed death of the fully differentiated cells (Figure 4). In fact, this increase was accompanied by an important decrease in cell viability as well as a diminution in CD14 expression (Figures 3(b) and 5(a)). Elsewhere, it is well established that, in hematopoietic tissues, apoptosis is coupled to terminal differentiation of myeloid progenitors, even though the mechanism responsible for the activation of apoptosis during myeloid maturation is still poorly understood [52, 53]. It is also noteworthy to mention that several reports have highlighted the role of caspases in the terminal differentiation of a variety of cell types [54]. One example is the differentiation of human blood monocytes into macrophages, a process that is blocked by synthetic caspase inhibitors [55]. It has been also reported that caspase 8 deletion in bone-marrow cells resulted in arrest of hemopoietic progenitor functioning and that its deletion in cells of the myelomonocytic lineage led to the arrest of differentiation into macrophages and consequently to cell death [56].

Among the differentially expressed genes in COLE treated cells (Table 3), genes encoding the mitogen-activated protein kinase kinase kinases (MAPKKKs) MAP3K2, MAP3K5, and MAP3K7 were highly expressed, while MAP2K5 and MAPK14/p38-*α* were downregulated. The MAPKKKs act at the upstream of the MAP kinase cascade which regulates important cellular processes such as gene expression, cell proliferation, differentiation, cell survival, and death. The MAP3K5 activates the c-Jun N-terminal kinase (JNK) and p38 mitogen-activated protein kinases [57] while the MAP3K2 is reported to preferentially activate JNK [58, 59]. However, the significant decrease in the MAPK14/p38-*α* may suggest that the effect of COLE on K562 cells is likely to be mediated by the JNK MAPKs, rather than the p38 MAPKs. JNK plays an important role in apoptosis pathways weather by activating proapoptotic or antiapoptotic genes. Particularly, it was shown that prolonged, but not transient, JNK activation promotes the cell death [60]. Our results showed that* JKAMP* gene which encodes for, a membrane-anchored regulator of the duration of JNK1 activity, was upregulated (1.54 ± 0.051). The elevated expression of JKAMP, as reported by Kadoya et al., [61], results in sustained JNK activity. These findings may indicate the potential role of JNK pathway in the COLE-induced apoptosis in K562 cells.

Thanks to their large regulatory domains, some MAPKKKs can interact with upstream regulators, have functions such as ubiquitylation, and be activated by relief of autoinhibition and oligomerization [62]. In this context, MAP3K2 has been reported to play an important role in NF-kappaB signaling pathway by activating the I-kappaB kinases which in turn phosphorylate the inhibitory factors of the nuclear factor kappaB (NF-kappaB) resulting in their rapid ubiquitination and the liberation of NF-kappaB complex which translocate from the cytoplasm to the nucleus [63]. MAP3K7, in association with other proteins, could also be required for the activation of NF-kappaB. According to the GO analysis (Table S1), COLE treatment upregulated genes implicated in NF-kappaB-related categories such as positive regulation of NF-kappaB transcription factor activity, I-kappaB kinase-NF-kappaB cascade and its positive regulation, and I-kappaB phosphorylation. Genes encoding the signal transducers in the NF-kappaB pathway, TRAF5, and TRAF6, as well as the activator of NF-kappaB signaling SNIP1, were upregulated. Interestingly, an increased expression of* NFKB1* gene, encoding for the precursor of the mature NF-kappaB p50, was detected in COLE treated cells compared to untreated K562 cells. NFKB is well recognized as a central activator of the antiapoptotic cascades in response to external stimuli or intrinsic immune reactions, and its prosurvival activity has been implicated in a variety of biological processes [60, 64]. In the B cell lineage, the activity of NF-*κ*B is required for the completion of various developmental stages including differentiation and the response of these cells to antigens [65]. NF-kappaB also plays an important role in the survival and development of T cells [66] and has been recently reported to mediate the differentiation of mesenchymal stem cells [67].

Several studies have demonstrated a crosstalk between the NF-kappaB and JNK pathways seen as an inhibitory effect of NF-kappaB on apoptosis through the suppression of JNK activity and that this suppressive effect may occur through different mechanisms [60]. This suggests that the balance between JNK and NF-kappaB activities is crucial to determine the cell fate, survival or death. We hypothesize here that COLE-induced apoptosis or differentiation is likely to be determined by the crosstalk between these two pathways in K562 cells. Further investigations regarding protein expression and activity are needed to confirm these findings.

## 5. Conclusions

Our study demonstrates for the first time that olive leaf extract exhibits an antileukemia effect on the human chronic myeloid leukemia cells. Olive leaf extract was shown to inhibit the proliferation of K562 cells by inducing cell cycle arrest, apoptosis, and differentiation toward the monocyte lineage. The induced expression of molecules involved in differentiation toward the monocyte/macrophage lineage as well as molecules related to apoptosis and cell cycle regulation confirmed these findings and provides insights into the mechanism by which olive leaf exhibits its antileukemia effect.

## Supplementary Material

The Supplementary Material provides the Gene Ontology analysis (GO analysis) of the differentially expressed genes after treatment with 150 µg/mL of COLE (Chemlali Olive Leaf Extract). Genes with more than 1.5 fold change in expression levels between control and 150 µg/mL of COLE treated cells, were classified into categories according to the cell biological processes. Table S1 represents the results of GO analysis of the up-regulated genes. Table S2 represents the results of GO analysis of the down-regulated genes.

## Figures and Tables

**Table 1:** Retention time and concentration of the main phenolic compounds present in Chemlali Olive Leaf Extract (COLE).

Peak number	Retention time (min)	Amount (mg/mL)	Compound
1	9.91	0.108	Hydroxytyrosol
2	15.225	0.047	Verbascoside
3	15.634	0.536	Luteolin-7-glucoside
4	17.344	0.529	Apigenin-7-glucoside
5	18.737	7.453	Oleuropein
6	22.127	0.089	Luteolin
7	25.056	0.012	Apigenin

**Table 2:** The distribution of cell cycle in K562 cells treated with Chemlali Olive Leaf Extract (COLE).

		Day 1	Day 2	Day 3	Day 4
G0/G1	Control	27.63 ± 2.90	24.50 ± 0.45	31.36 ± 1.85	29.83 ± 3.52
	100 µg/mL	27.36 ± 4.07	22.56 ± 2.67	27.76 ± 2.09	23.45 ± 3.81
	150 µg/mL	39.80 ± 2.26*	31.01 ± 1.3**	20.96 ± 2.78**	12.43 ± 0.64**

S	Control	22.07 ± 1.15	21.07 ± 1.33	17.70 ± 2.1	20.00 ± 1.6
	100 µg/mL	27.69 ± 2.59*	22.35 ± 1.83	18.93 ± 0.72	16.15 ± 0.73*
	150 µg/mL	14.65 ± 3.58*	24.54 ± 0.6*	18.49 ± 3.47	16.88 ± 2.59

G2/M	Control	33.57 ± 0.98	30.20 ± 1.44	27.93 ± 1.36	28.60 ± 0.7
	100 µg/mL	24.41 ± 2.27**	36.97 ± 1.6**	32.47 ± 1.1*	36.11 ± 2.64**
	150 µg/mL	18.93 ± 0.54**	27.72 ± 2.07	37.25 ± 2.1**	34.00 ± 1.83**

Results are represented as the means ± SD of three independent experiments.

∗ and ∗∗ mean that difference between control and treated cells in each phase (G0/G1, S and G2/M) is statistically significant at *P* < 0.05 and *P* < 0.01, respectively.

**Table 3:** Changes in gene expression profile induced by treatment Of K562 cells with Chemlali Olive Leaf Extract (COLE).

Gene symbol	Gene title	Accession number	Molecular function/biological process	Gene expression (treatment/control)			
				100 µg/mL		150 µg/mL	
				Fold change	*P* value	Fold change	*P* value
CHEK2	CHK2 checkpoint homolog (*S. pombe*)	NM_001005735 /// NM_007194 /// NM_145862	Cell cycle, kinase activity	1.702	1.03*E* − 02	1.620	1.44*E* − 02

CDC25C	Cell division cycle 25 homolog C (*S. pombe*)	NM_001790 /// NM_022809	Cell cycle, regulation of cyclin-dependent protein kinase activity	−1.452	3.98*E* − 02	−1.553	2.40*E* − 02

CDC25A	Cell division cycle 25 homolog A (*S. pombe*)	NM_001789 /// NM_201567	Phosphoprotein phosphatase activity, regulation of cyclin-dependent protein kinase activity	2.284	8.1*E* − 03	2.324	7.60*E* − 03

CASP6	Caspase 6, apoptosis-related cysteine peptidase	NM_001226 /// NM_032992	Positive regulation of apoptosis, acute inflammatory response to nonantigenic stimulus, apoptosis, hydrolase activity, induction of apoptosis, proteolysis, and peptidase activity	1.705	3.64*E* − 02	1.779	2.88*E* − 02

DFFA	DNA fragmentation factor, 45 kDa, alpha polypeptide	NM_004401 /// NM_213566	Apoptosis, induction of apoptosis, caspase-activated deoxyribonuclease activity, DNA fragmentation involved in apoptosis, positive regulation of apoptosis, and negative regulation of apoptosis	2.678	1.30*E* − 02	1.967	4.30*E* − 02

BID	BH3 interacting domain death agonist	NM_001196 /// NM_197966 /// NM_197967	Positive regulation of apoptosis, death receptor binding, release of cytochrome c from mitochondria, activation of proapoptotic gene products, and regulation of mitochondrial membrane permeability	1.644	3.46*E* − 02	1.577	4.48*E* − 02

CASP8	Caspase 8, apoptosis-related cysteine peptidase	NM_001080124 /// NM_001080125 /// NM_001228 /// NM_033355 /// NM_033356 /// NM_0	Peptidase activity, proteolysis, apoptosis, regulation of apoptosis, positive regulation of I-kappaB kinase-NF-kappaB cascade, activation of proapoptotic gene products, induction of apoptosis by extracellular signals, and macrophage differentiation	1.310	1.3*E* − 01	1.633	2.63*E* − 02

IGF1R	Insulin-like growth factor 1 receptor	NM_000875	Positive regulation of proliferation, positive regulation of migration, and antiapoptosis	−2.397	3.09*E* − 02	−3.312	1.10*E* − 02

HSPA5	Heat shock 70 kDa protein 5	NM_005347	Negative regulation of caspase activity, caspase inhibitor activity	−1.790	2.10*E* − 03	−2.454	0.4*E* − 03

BCL2	B cell CLL/lymphoma 2	NM_000633 /// NM_000657	Activation of proapoptotic gene products, negative regulation of myeloid cell apoptosis, regulation of programmed cell death, and negative regulation of mitotic cell cycle	−1.782	1.13*E* − 02	−1.804	1.05*E* − 02

FECH	Ferrochelatase	NM_000140 /// NM_001012515	Erythrocyte differentiation	−1.657	3.60*E* − 02	−1.975	1.38*E* − 02

GYPA	Glycophorin A (MNS blood group)	NM_002099	Erythrocyte differentiation	−3.823	8.40*E* − 03	−4.227	6.5*E* − 03

HBE1	Hemoglobin, epsilon 1	NM_005330	Erythrocyte differentiation	−2.678	2.30*E* − 02	−3.419	1.10*E* − 02

NFE2	Nuclear factor (erythroid-derived 2), 45 kDa	NM_001136023 /// NM_006163	Regulation of transcription, cell-cell signaling, and megakaryocyte differentiation	−1.611	2.12*E* − 01	−1.366	3.86*E* − 01

TUBB1	Tubulin, beta 1	NM_030773	Microtubule-based process	−1.565	7.53*E* − 02	−1.4501	1.18*E* − 01

BACH2	BTB and CNC homology 1, basic leucine zipper transcription factor 2	NM_001170794 /// NM_021813	Regulation of transcription, DNA dependent	−1.569	1.21*E* − 02	−1.798	0.47*E* − 02

ACIN1	Apoptotic chromatin condensation inducer 1	NM_001164814 /// NM_001164815 /// NM_001164816 /// NM_001164817 /// NM_014977	Positive regulation of monocyte differentiation, apoptosis, apoptotic chromosome condensation, and ATPase activity	1.403	3.65*E* − 02	1.669	9.47*E* − 03

IFI16	Interferon, gamma-inducible protein 16	NM_005531	Monocyte differentiation, myeloid cell differentiation, regulation of transcription, DNA dependent, DNA damage response, and cell proliferation	1.400	4.75*E* − 02	2.186	2.80*E* − 03

EGR1	Early growth response 1	NM_001964	Transcription factor activity, regulation of transcription, DNA dependent	1.441	1.11*E* − 01	1.765	3.38*E* − 02

NFYA	Nuclear transcription factor Y, alpha	NM_002505 /// NM_021705	Transcription factor activity	1.916	0.95*E* − 03	1.729	1.85*E* − 03

FOXP1	Forkhead box P1	NM_001012505 /// NM_032682	Negative regulation of transcription, transcription repressor activity	−1.376	3.91*E* − 02	−1.552	0.0141

IL8	Interleukin-8	NM_000584	Immune response, neutrophil chemotaxis, regulation of cell adhesion, neutrophil activation, negative regulation of cell proliferation, cell cycle arrest, inflammatory response, and chemotaxis	1.015	9.62*E* − 01	3.638	1.46*E* − 02

CXCL2	Chemokine (C-X-C motif) ligand 2	NM_002089	Neutrophil chemotaxis, immune response, inflammatory response, and chemotaxis	1.559	1.71*E* − 01	5.831	2.74*E* − 03

CXCL3	Chemokine (C-X-C motif) ligand 3	NM_002090	Neutrophil chemotaxis, immune response, inflammatory response, chemotaxis, and leukocyte chemotaxis	1.447	2.75*E* − 01	5.181	4.94*E* − 03

NUP85	Nucleoporin 85 kDa	NM_024844	Macrophage chemotaxis, cytokine-mediated signaling pathway, and chemotaxis	1.740	0.54*E* − 02	1.636	8.33*E* − 03

AP1G1	Adaptor-related protein complex 1, gamma 1 subunit	NM_001030007 /// NM_001128	Microtubule cytoskeleton organization, intracellular protein transport, endocytosis, and vesicle mediated transport	1.543	0.58*E* − 02	1.547	5.69*E* − 03

CTNNB1	Catenin (cadherin-associated protein), beta 1, 88 kDa	NM_001098209 /// NM_001098210 /// NM_001904	Wnt receptor signaling pathway through beta-catenin, cell morphogenesis involved in differentiation, regulation of transcription, regulation of cell differentiation, cadherin, positive regulation of MAPKKK cascade, cell-cell adhesion, cell-matrix adhesion, regulation of cell adhesion, hemopoiesis, and so forth	1.449	1.29*E* − 02	1.530	8.09*E* − 03

ICAM3	Intercellular adhesion molecule 3	NM_002162	Cell-cell adhesion, integrin binding	1.841	3.0*E* − 03	1.775	3.76*E* − 03

PNN	Pinin, desmosome associated protein	NM_002687	Cell-cell adhesion, negative regulation of cell cycle	1.746	1.43*E* − 02	2.061	5.79*E* − 03

HSPB11	Heat shock protein family B (small), member 11	NM_016126	Cell adhesion	1.640	0.48*E* − 02	1.709	3.58*E* − 03

RAB21	RAB21, member RAS oncogene family	NM_014999	Protein transport, endocytosis	1.559	0.37*E* − 02	1.605	2.95*E* − 03

RAB5C	RAB5C, member RAS oncogene family	NM_004583 /// NM_201434	Protein transport, endocytosis	1.821	1.10*E* − 02	1.844	1.03*E* − 02

RAB11A	RAB11A, member RAS oncogene family	NM_004663	Protein transport, endocytosis	1.861	1.98*E* − 02	1.964	1.51*E* − 02

MAP3K2	Mitogen-activated protein kinase kinase kinase 2	NM_006609	Activation of MAPK activity, activation of JUN kinase activity, cell proliferation, and protein amino acid phosphorylation	1.290	2.04*E* − 01	1.956	1.63*E* − 02

MAP3K5	Mitogen-activated protein kinase kinase kinase 5	NM_005923	MAPKKK cascade, activation of JUN kinase activity, protein amino acid phosphorylation, activation of MAPK activity, and apoptosis	1.836	0.52*E* − 02	1.554	1.59*E* − 02

MAP3K7	Mitogen-activated protein kinase kinase kinase 7	NM_003188 /// NM_145331 /// NM_145332 /// NM_145333	I-kappaB phosphorylation, activation of NF-kappaB-inducing kinase activity, MAPKKK cascade, positive regulation of JNK cascade, T cell receptor signaling pathway, negative regulation of apoptosis, and regulation of interleukin-2 production	1.423	2.41*E* − 02	1.575	1.04*E* − 02

TRAF6	TNF receptor-associated factor 6	NM_004620 /// NM_145803	Positive regulation of interleukin-12 biosynthetic process, regulation of apoptosis, protein polyubiquitination, T cell receptor signaling pathway, and positive regulation of I-kappaB kinase-NF-kappaB cascade	1.409	3.10*E* − 02	1.506	1.76*E* − 02

NFKB1	Nuclear factor of kappa light polypeptide gene enhancer in B cells 1	NM_001165412 /// NM_003998	Inflammatory response, regulation of transcription, apoptosis, regulation of lipid metabolic process, and positive regulation of foam cell differentiation	1.498	0.11*E* − 02	1.695	0.42*E* − 03

SNIP1	Smad nuclear interacting protein 1	NM_024700	Regulation of transcription, insulin receptor signaling pathway, and I-kappaB kinase-NF-kappaB cascade	1.683	3.09*E* − 02	1.828	1.94*E* − 02

MAPK14	Mitogen-activated protein kinase 14	NM_001315 /// NM_139012 /// NM_139013 /// NM_139014	Stress-activated MAPK cascade, cell surface receptor linked signal transduction, skeletal muscle tissue development, Ras protein signal transduction, response to stress, angiogenesis, and so forth	−1.468	1.52*E* − 02	−1.733	4.33*E* − 03

MAP2K5	Mitogen-activated protein kinase kinase 5	NM_002757 /// NM_145160	Regulation of cell growth, protein amino acid phosphorylation, MAPKKK cascade, and signal transduction	−1.656	0.93*E* − 02	−1.504	1.90*E* − 02

Microarray analysis was performed at 3rd day of treatment on pooled RNAs from control cells and cells treated with 100 and 150 µg/mL of COLE from 2 independent experiments.
